# High-κ Dielectric on ReS_2_: In-Situ Thermal Versus Plasma-Enhanced Atomic Layer Deposition of Al_2_O_3_

**DOI:** 10.3390/ma12071056

**Published:** 2019-03-30

**Authors:** Ava Khosravi, Rafik Addou, Massimo Catalano, Jiyoung Kim, Robert M. Wallace

**Affiliations:** 1 Department of Materials Science and Engineering, The University of Texas at Dallas, Richardson, TX 75080, USA; axk149530@utdallas.edu (A.K.); rafik.addou@oregonstate.edu (R.A.); mxc160230@utdallas.edu (M.C.); jiyoung.kim@utdallas.edu (J.K.); 2 School of Chemical, Biological, and Environmental Engineering, Oregon State University, Corvallis, OR 93771, USA; 3 Institute for Microelectronics and Microsystems, National Council for Research (IMM-CNR), Via Monteroni, ed. A3, 73100 Lecce, Italy

**Keywords:** rhenium sulfide, atomic layer deposition, plasma-enhanced atomic layer deposition, X-ray photoelectron spectroscopy

## Abstract

We report an excellent growth behavior of a high-κ dielectric on ReS_2_, a two-dimensional (2D) transition metal dichalcogenide (TMD). The atomic layer deposition (ALD) of an Al_2_O_3_ thin film on the UV-Ozone pretreated surface of ReS_2_ yields a pinhole free and conformal growth. In-situ half-cycle X-ray photoelectron spectroscopy (XPS) was used to monitor the interfacial chemistry and ex-situ atomic force microscopy (AFM) was used to evaluate the surface morphology. A significant enhancement in the uniformity of the Al_2_O_3_ thin film was deposited via plasma-enhanced atomic layer deposition (PEALD), while pinhole free Al_2_O_3_ was achieved using a UV-Ozone pretreatment. The ReS_2_ substrate stays intact during all different experiments and processes without any formation of the Re oxide. This work demonstrates that a combination of the ALD process and the formation of weak S–O bonds presents an effective route for a uniform and conformal high-κ dielectric for advanced devices based on 2D materials.

## 1. Introduction

Transition metal dichalcogenides (TMDs) have emerged as strong candidates to be implemented as the semiconductor channel in Field Effect Transistor (FET) applications [1,2]. Their remarkable properties, such as sizable electronic bandgaps and the capability to scale down to sub-nanometer thickness, make semiconducting TMDs ideal candidates for applications in nanoelectronics [3]. Most semiconducting TMDs show significant changes in electronic properties from the bulk to the monolayer form, such as an indirect to direct band gap crossover [4]. Such properties provide opportunities to engineer material heterostructures to fit specific device requirements.

The reports on ReS_2_ are relatively limited compared to other TMD materials. ReS_2_ crystallizes in a distorted 1T phase, unlike most commonly studied TMDs, and this crystal structure causes unusual electronic and vibrational decoupling. Interestingly, the band gap type of ReS_2_ in monolayer and bulk form remains uncertain. ReS_2_ has been reported to have a direct bandgap, regardless of its thickness [5]. The ReS_2_ direct bandgap is reported to be 1.35 eV in bulk and 1.42 eV in its monolayer form [6,7]. Recent computational and experimental studies address controversies on the band gap of ReS_2_ [8,9,10,11]. Unlike early literature reports, such calculations revealed a layer dependence of the valence band maximum (VBM) position in ReS_2_ and indirect band gap character for bulk ReS_2_ [12,13,14]. Recently, ReS_2_-based FET devices have attracted interest [15,16,17,18,19,20,21,22], with a room temperature I_on_/I_off_ ratio on the order of ~10^5^ using few-layer ReS_2_ flakes and Al_2_O_3_ as the high-κ top-gate dielectric [15]. In another report, top-gate FETs based on few-layer ReS_2_ nanosheets encapsulated in an Al_2_O_3_ dielectric showed I_on_/I_off_ > 10^6^ [16]. Also, a high-performance ReS_2_-based tunneling FET was reported to be exploiting an O_2_ plasma treatment to accurately control the thickness of ReS_2_ [18]. Such encouraging results suggest that ReS_2_ could be a very promising candidate for future nanoelectronics.

When integrating TMDs into nanoelectronic devices, an optimized dielectric–semiconductor interface is crucial for TMD-based devices to reach commercial viability [23]. Previous reports demonstrate reduced Coulombic scattering and improved electron field-effect mobility in the TMD channel when interfacing with a high-κ dielectric [24]. Additionally, a conformal, pinhole-free dielectric layer inhibits the leakage current in a TMD-based device. However, the deposition of high-quality high-κ dielectrics onto the surface of TMD semiconductors has been a challenge due to the relative dearth of dangling bonds. The lower dangling bond density on the TMD surface causes the direct atomic layer deposition (ALD) of high-κ dielectrics to form islands instead of a continuous film [25]. The deposition of various dielectrics on 2D materials such as graphene, black phosphorus, MoS_2_, and WSe_2_ has been extensively studied [25,26,27,28,29,30,31]; however, a detailed interface investigation of a high-κ dielectric on ReS_2_ and ReSe_2_ remains of interest. Although a high-κ dielectric has been used for ReS_2_ based devices [15,16,19], the ReS_2_ interface chemistry with Al_2_O_3_ has not been reported. In this work, thermal ALD and PEALD (plasma-enhanced atomic layer deposition) are compared in order to study the high-κ dielectric interfacial chemistry. In-situ half-cycle X-ray photoelectron spectroscopy and ex-situ atomic force microscopy (AFM) and Raman have been used to explore this interface.

## 2. Materials and Methods

Bulk ReS_2_ crystals purchased from HQ Graphene (Groningen, The Netherlands) were used to prepare clean surfaces by mechanical exfoliation using Scotch^(R)^ Magic^TM^ tape (3M, Maplewood, MN, USA). Samples were loaded into an ultra-high vacuum (UHV) system, with 5 min of air exposure for an ALD/PEALD of Al_2_O_3_ and corresponding in-situ X-ray photoelectron spectroscopy (XPS, Sienta Omicron GmbH, Taunusstein, Germany) analysis. The UHV apparatus (base pressure ~10^−10^ mbar) and XPS employed in this work are described in detail elsewhere [32]. Samples were annealed at 250 °C in a chamber (base pressure ~10^−9^ mbar) under UHV for 1 h prior to the ALD processes, to desorb the adventitious carbon and oxygen from the sample surface (Figure S1a,b). After annealing, the surface chemistry of the sample did not change (Figure S1c,d). The UHV system was connected to the PEALD chamber (Picosun PR200, Espoo, Finland) through an intermediate chamber. A trimethylaluminum (TMA) and H_2_O (pulse time of 0.1 s and a 4.0 s Ar purge) were used for the thermal ALD process. During the PEALD process, TMA with a 0.1 s pulse time and a 4.0 s Ar purge, and a remote O_2_ plasma with a 3.0 s pulse and 4.0 s Ar purge were used as precursors. A Litmas RF-plasma source (RF power 2000 W) with an O_2_ gas flow of 120 sccm generated the remote O_2_ plasma. The deposition chamber (base pressure of 3 mbar) was held at 200 °C throughout all deposition processes of this work. The nucleation and the growth of Al_2_O_3_ by ALD and PEALD was studied by means of an in-situ half-cycle process. The XPS scans were measured following each successive half cycle, up to five full cycles and following 10, 40, and 80 full ALD/PEALD cycles (Figure S2). Each XPS spectra was recorded using a monochromatic Al Kα x-ray source with an Omicron EA125 hemispherical analyzer (Sienta Omicron GmbH, Taunusstein, Germany, pass energy of a 15 eV and a resolution of ±0.05 eV). AAnalyzer Version 1.36 software was used to deconvolute the XPS data [33]. An ex-situ atomic force microscope (AFM, Veeco Model 3100 Dimension V, Veeco, Plainview, NY, USA), operating in the tapping mode under ambient conditions, was also used to characterize the surface morphology after 80 full cycles of each process, and WSxM was used to analyze the AFM images [34]. An ex-situ Raman measurement was also acquired by a 532 nm wavelength laser with 0.22 mW power and ~500 nm spot size. The atomic Scanning Transmission Electron Microscope High-Angle Annular Dark-Field (STEM HAADF) images were obtained using a JEOL ARM200F microscope (JEOL, Tokyo, Japan) equipped with a spherical aberration (Cs) corrector (CEOS GmbH, Heidelberg, Germany) and operated at 200 kV. The corrector was carefully tuned by the Zemlin tableau method with Cs¼ 0.5 lm, and the resolution was demonstrated to be around 0.1 nm.

## 3. Results and Discussion

### 3.1. Surface Analysis of the Exfoliated ReS_2_ Surface

**Figure 1:**
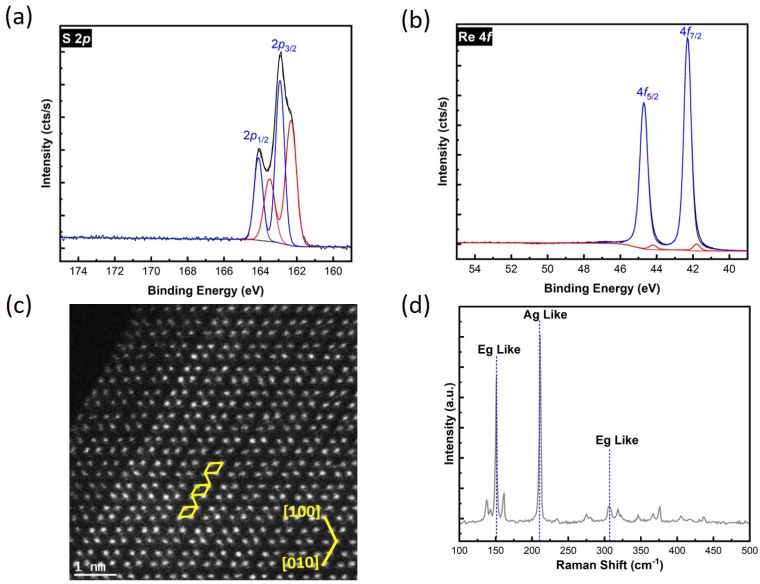
(**a**) S 2*p* and (**b**) Re 4*f* X-ray photoelectron spectroscopy (XPS) core level spectra obtained on a freshly exfoliated ReS_2_ surface. The additional component in the lower binding energy is shown in red for both spectra. (**c**) High-angle annular dark field (HAADF) presents only Re atoms and (**d**) Raman spectrum of exfoliated ReS_2_.

Figure 1a,b show the S 2*p* and Re 4*f* core level spectra, measured on an as-exfoliated ReS_2_ surface. It is important to note that we found two distinct states (red and blue spectra) in the S 2*p* core level in all samples studied in this work. These states are separated by 0.6 eV, and the ratio between their areas is 0.7. The binding energy of the S 2*p*_3/2_ core level of elemental sulfur (S^0^) is reported to be ~164.0 eV, which was not detected here [35]. Since there is no chemical state detected in the O 1*s* core level at ~530 eV (Figure S1b), the additional sulfur chemical state cannot be assigned to the formation of a S–O bond [26]. The same S 2*p* core level spectrum line shape has been shown in previous XPS reports of ReS_2_ [36,37,38,39,40,41]; however, the S 2*p* doublets were not examined carefully, and a second state was not detected. Other reports on distorted 1T-ReS_2_ show a similar binding energy separation (0.6 eV) in the S 2*p* core level [39,40]. An additional state in the Re 4*f* core level was detected at a 0.6 eV lower binding energy than the most intense peak for all samples used in this study (red spectra, Figure 1b). The metallic Re (Re^0^) state appears at a 1.1 eV lower binding energy from the Re^4+^ chemical state however, the binding energy separation between the two Re 4*f* doublets detected here is smaller (red and blue curves, Figure 1b) [40]. The additional state is not associated with the Re–O chemical state, as Re–O should be detected at a higher binding energy [42]. This extra feature at the lower binding energy in the Re 4*f* core level could be the result of surface roughness or defects [43]. The separation between the S 2*p*_5/2_ and Re 4*f*_7/2_ core levels for the distorted 1T-ReS_2_ phase (blue spectra, Figure 1) was 120.4 eV, in agreement with the previous report [44]. Interestingly, the S:Re ratio is 2:1 when considering the integrated area of both states (both red and blue spectra) in the S 2*p* and Re 4*f* core levels, while the S:Re ratio is only 1:1 when considering only the high binding energy state in the S 2*p* core level (blue spectra). The extra features at both S 2*p* and Re 4*f* were detected at different spots on the same sample, as well as on the same sample following a second exfoliation.

A flake from an exfoliated ReS_2_ sample was imaged using STEM, as shown by the plan view in Figure 1c. ReS_2_ has triclinic symmetry in the crystal structure, wherein Re atoms in the layer form a zigzag Re–Re chain [17,20,45]. Figure 1c provides a view of the crystalline structure of the ReS_2_, and shows that the four Re atoms are arranged in a diamond-like shape and form atomic chains. The atomic structure confirms the distorted 1T phase, as suggested by previous investigations [20,36,45]. The TEM data show the presence of only one dominant distorted 1T phase in the ReS_2_ flakes. To further study the crystal structure of ReS_2_, Raman spectroscopy was performed on as-exfoliated bulk ReS_2_, showing conclusive evidence of having a distorted 1T structure. Because of the low symmetry of the distorted 1T phase, 18 Raman peaks were found in the range of 100–500 cm^−1^ in bulk ReS_2_, consistent with previous studies [46,47]. The primary Raman active modes at 150.2 cm^−1^ and 211.0 cm^−1^, associated with Eg-like and Ag-like modes, are presented in Figure 1d [48,49].

#### Thermal ALD of Al_2_O_3_ on ReS_2_

**Figure 2:**
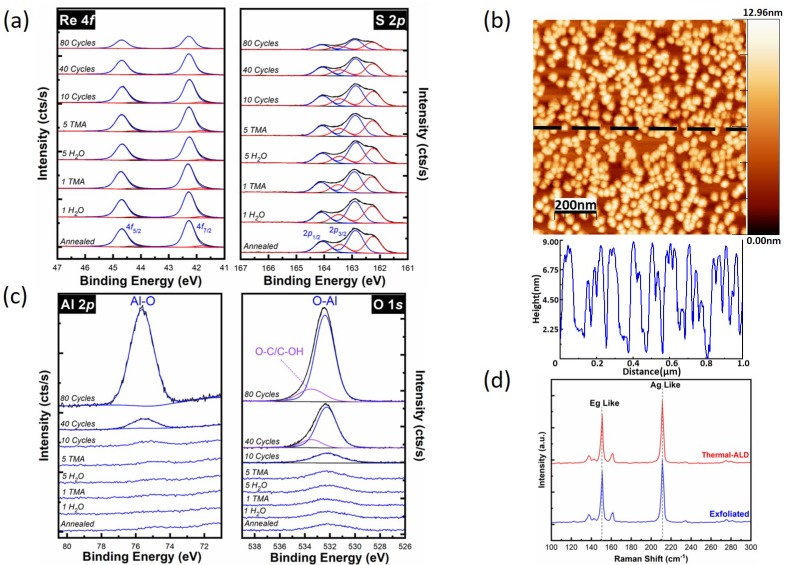
Thermal atomic layer deposition (ALD) of Al_2_O_3_ on ReS_2_. (**a**) The evolution of Re 4*f* and S 2*p* core level spectra, (**b**) Ex-situ AFM image and line profile recorded following 80 cycles ALD of Al_2_O_3_, (**c**) Al 2*p* and O 1*s* core level spectra up to 80 cycles of Al_2_O_3_, and (**d**) Raman spectra comparison between exfoliated ReS_2_ and following 80 cycles of ALD of Al_2_O_3_.

The evolution of the Re 4*f* and S 2*p* core levels throughout the thermal ALD of Al_2_O_3_ is shown in Figure 2a. The Re 4*f* and S 2*p* core level spectra were obtained from the ReS_2_ surface after in-situ annealing, after the first successive half-cycle of TMA and H_2_O, after the fifth half-cycle of TMA and H_2_O, and following 10, 40, and 80 full cycles of Al_2_O_3_. Only the XPS of the first and fifth half-cycle exposures are shown, as no significant changes occurred in the surface chemistry in between these measurements. Figure 2a indicates that the ReS_2_ sample is free of any additional chemical states in the Re 4*f* or S 2*p* core levels throughout the thermal ALD process, other than the state discussed above (Figure 1a,b), and rhenium oxide or sulfur oxide compounds (Re–O, Re–O–S, or S–O bonds) are below the XPS detection limit. The acquisition of XPS spectra for the Re 4*f* and S 2*p* core levels were extended on the higher binding energy side up to 55 eV and 175 eV (Figure 1a,b), respectively, to ensure that there were no additional chemical states at the Re 4*f* and S 2*p* core level spectra. Depositing Al_2_O_3_ on ReS_2_ decreases the intensities of the Re 4*f* and S 2*p* core levels (Figure 2a) due to XPS signal attenuation. However, the Re 4*f* and S 2*p* core levels are still clearly detectable even after 80 full cycles of thermal ALD due to the clustering of the Al_2_O_3_ film, as suggested by the ex-situ AFM topographic image in Figure 2b. At the initial stage, the slow growth rate indicates that thermal ALD of Al_2_O_3_ on the untreated ReS_2_ surface requires 10 thermal ALD cycles to nucleate, displaying the *substrate-inhibited* growth.

Figure 2b shows a topographic image obtained by AFM of Al_2_O_3_ on ReS_2_ following 80 full cycles employing the thermal ALD process. After the thermal ALD on ReS_2_, an islanding growth is observed because of the relative dearth of dangling bonds on 2D TMD surfaces [25,26]. The AFM line profile shows that the height of these Al_2_O_3_ islands is ~8 nm, with a root mean square (RMS) surface roughness of 2.7 nm.

The corresponding Al 2*p* and O 1*s* core level spectra measured during the thermal ALD process are shown in Figure 2c. Aluminum remains near the XPS detection limit in the Al 2*p* core level following Al_2_O_3_ deposition up to 10 thermal ALD cycles. After 10 full cycles of Al_2_O_3_, the peak at 75.4 eV caused by the formation of Al–O bonds starts to increase, with an increasing number of ALD cycles. The analysis of this peak in the Al 2*p* core level after 40 and 80 full cycles reveals the formation of an Al_2_O_3_ film on ReS_2_. The line shape of the background intensity at the Al 2*p* core level increases at lower binding energies due to the presence of a Re 4*f* loss feature at 68 eV (Figure S3). After annealing, a small amount of oxygen (7.0 atomic%) was still detectable on the surface of the ReS_2_ sample (Figure 2c). The O 1*s* core level shown in Figure 2c after 40 and 80 full ALD cycles is deconvoluted into two peaks. The peak at 532.3 eV is associated with the Al−O chemical state and the peak at the higher binding energy (533.5 eV) is attributed to intermediate species, such as carbonyl and formate [28,50].

ReS_2_ samples characterized via ex-situ Raman spectroscopy following thermal ALD are consistent with the Raman spectra recorded on an as-exfoliated ReS_2_ surface [46,47]. Amorphous Al_2_O_3_ on the surface does not affect the Raman spectra of the substrate, and therefore structural changes through different processes of this work should be observable. Figure 2d shows the Raman spectra from as-exfoliated ReS_2_ and after 80 cycles thermal ALD of Al_2_O_3_. Two prominent Raman peaks at 150.2 cm^−1^ and 211.0 cm^−1^ are assigned to E_g_-like and A_g_-like vibrational modes, respectively [51]. In TMDs, Raman active modes have been reported to shift with changes in strain [52], temperature [53], and doping [54]. Figure 2d confirms that ReS_2_ remains unaffected following the annealing and the ALD process.

### 3.2. Plasma-Enhanced ALD of Al_2_O_3_ on ReS_2_

**Figure 3:**
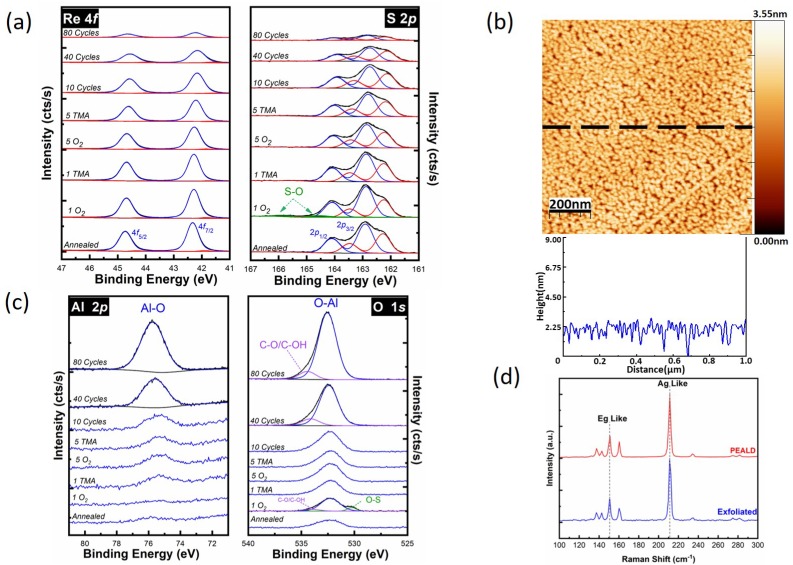
Plasma-enhanced atomic layer deposition (PEALD) of Al_2_O_3_ on ReS_2_. (**a**) The evolution of (**a**) Re 4*f* and S 2*p* core level spectra, (**b**) Ex-situ AFM image and line profile recorded following 80 cycles PEALD of Al_2_O_3_, (**c**) Al 2*p* and O 1*s* core level spectra up to 80 cycles of Al_2_O_3_, and (**d**) Raman spectra recorded on exfoliated ReS_2_ and after 80 cycles of PEALD of Al_2_O_3_.

PEALD has been reported to achieve conformal high-κ dielectrics on the TMD surface without utilizing any surface pretreatment [55]. Based on this study, PEALD is used to improve the coverage of deposited Al_2_O_3_ on ReS_2_. More importantly, this work studies the interfacial chemistry changes during the PEALD process on ReS_2_. Similar to the thermal ALD process, an in-situ half-cycle PEALD study was performed to monitor the chemistry of the Al_2_O_3_/ReS_2_ interface. Figure 3a shows the Re 4*f* and S 2*p* core level spectra obtained from the annealed surface, after the first half-cycle of O_2_ plasma and TMA, after the fifth half-cycles of O_2_ plasma and TMA, and following 10, 40, and 80 full cycles of Al_2_O_3_. No additional chemical states were detected in the Re 4*f* or S 2*p* core levels on the surface of the annealed ReS_2_ sample. After the first pulse of remote O_2_ plasma, an additional chemical state in the corresponding S 2*p* core level was detected at 164.8 eV (Figure 3a, green peaks), which was concurrent with the appearance of an additional chemical state at a lower binding energy (530.5 eV) in the corresponding O 1*s* core level in Figure 3c.

This additional chemical state corresponds to the S–O bonds created by the remote O_2_ plasma pulse. We estimated an oxidation of ~4.0% of S atoms on the surface after the first O_2_ plasma pulse. The S–O chemical bonds on the ReS_2_ surface act as nucleation sites, making the surface more reactive with the precursors. Through ligand exchange reactions, the S–O bonds are cleaned up by the successive TMA pulse and fall below the XPS detection limit [56]. During the rest of the PEALD process, the S–O chemical state remains below the XPS detection limit, and the ReS_2_ surface is free of any additional chemical states in the Re 4*f* or S 2*p* core levels, consistent with an abrupt interface. The first noticeable observation is that depositing Al_2_O_3_ on ReS_2_ using PEALD significantly decreases the intensities of the Re 4*f* and S 2*p* core levels from the substrate after 80 full cycles of PEALD, in comparison to thermal ALD (Figure 2a), which suggests better Al_2_O_3_ coverage on ReS_2_. This is consistent with the corresponding AFM topographic image in Figure 3b. The difference in thermal ALD and PEALD deposition after 80 cycles could be explained with the creation of S–O bonds as a reactive nucleation center in the PEALD method. Figure 3b shows the surface morphology of the sample after 80 PEALD cycles of Al_2_O_3_. The PEALD process results in a better coverage of the Al_2_O_3_ film with an RMS surface roughness of 0.49 nm compared to the thermal ALD process. The XPS-estimated Al_2_O_3_ thickness was 2.3 nm, which was calculated based on the substrate signal attenuation of the S 2*p* core level integrated intensity (Supplementary Materials, Section 3). The thickness calculated by XPS is underestimated due to the detection of the substrate signal through pinholes in the oxide film.

Figure 3c shows the evolution of the Al 2*p* and O 1*s* core level spectra of the PEALD process. Unlike the thermal ALD process, a small Al 2*p* peak is detected at 75.4 eV after the first TMA pulse, suggesting that, with the PEALD process, Al is deposited on the ReS_2_ surface without any inhibition time. The peak associated with the Al–O chemical state in Al 2*p* and O 1*s* core level spectra keeps increasing throughout the PEALD process. Similar to the thermal ALD process, the O 1*s* core level is deconvoluted into two main components of an Al–O chemical state at a low binding energy and an intermediate species chemical state at a higher binding energy. The ReS_2_ structure was monitored via ex-situ Raman spectroscopy before and after the PEALD (Figure 3d). The two prominent Raman peaks—E_g_-like at 150.2 cm^−1^ and A_g_-like at 211.0 cm^−1^—do not show any noticeable changes, confirming the stability of the ReS_2_ structure under the remote O_2_ plasma exposure of PEALD process.

The PEALD method results in a better coverage and lower root mean square (RMS) surface roughness due to a better nucleation and thus a thicker Al_2_O_3_ layer, as suggested by the associated XPS and AFM measurements. The PEALD of Al_2_O_3_ is greatly improved the low coverage and clustering behavior associated with the thermal ALD of Al_2_O_3_ on ReS_2_, however, pinholes with ~2 nm depth are observed, based on AFM measurements. Despite the improvement in Al_2_O_3_ coverage achieved with the PEALD process via the creation of S–O bonds, neither thermal ALD or PEALD yield conformal, pinhole-free Al_2_O_3_ on ReS_2_.

### 3.3. Al_2_O_3_ on A UV-Ozone Exposed ReS_2_ Surface 

**Figure 4:**
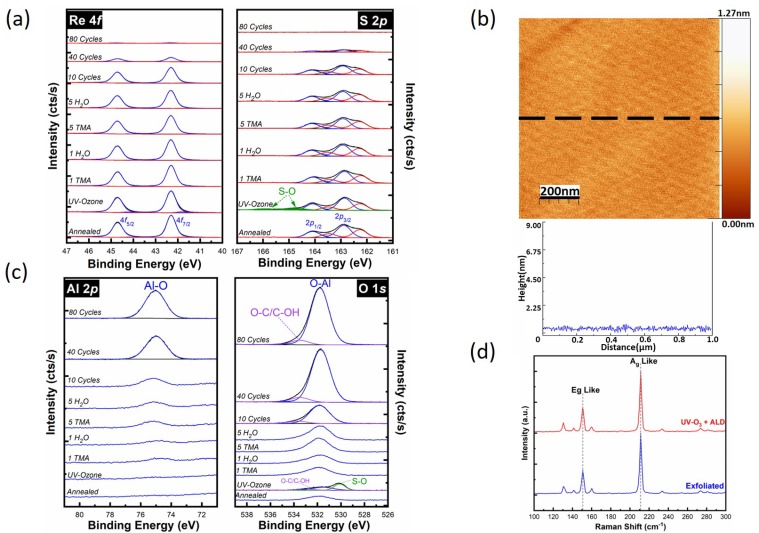
Deposition of Al_2_O_3_ using thermal ALD on functionalized ReS_2_ surface by UV-Ozone exposure. (**a**) The evolution of Re 4*f* and S 2*p* core level spectra, (**b**) Ex-situ AFM image and line profile recorded following 80 cycles PEALD of Al_2_O_3_, (**c**) Al 2*p* and O 1*s* core level spectra up to 80 cycles of Al_2_O_3_, and (**d**) Raman spectra from exfoliated ReS_2_ and after 80 cycles of Al_2_O_3_ on UV-Ozone treated ReS_2_.

To grow a uniform dielectric layer on TMDs, a number of surface functionalization methods were proposed, such as UV-Ozone pre-treatment [26,57], oxygen plasma pre-treatment [58], the use of ozone as the oxidant precursor during the ALD of Al_2_O_3_ [28], and metal-oxide seed layers [59]. Based on the PEALD results of this work on ReS_2_ and the previous study on MoS_2_ [26,60], the formation of S–O in sulfur-based TMD materials seems to be a promising route for surface functionalization. To further improve the surface coverage of Al_2_O_3_ on the ReS_2_, the sample is exposed to UV-Ozone as a surface pretreatment to form S–O bonds, and then subjected to the standard thermal ALD process. An in-situ study of the UV-Ozone exposure of ReS_2_ was performed and chemical state changes were monitored by XPS. The UV photons from a low-pressure mercury lamp were used to produce ozone in a chamber (base pressure of ~10^−9^ mbar) which was filled with O_2_ to a pressure of 900 mbar [61]. Then, the sample was transferred in-situ to the ALD chamber for Al_2_O_3_ deposition with TMA and H_2_O. Figure 4a shows the Re 4*f* and S 2*p* core level spectra obtained from the annealed surface after 15 min UV-Ozone exposure, after the first successive half-cycle of H_2_O and TMA, after the fifth half-cycle of H_2_O and TMA, and following 10, 40, and 80 full cycles of Al_2_O_3_. After UV-Ozone exposure, an additional chemical state is detected in the S 2*p* core level at 164.8 eV (Figure 4a), which suggests the formation of S–O bonds [26]. The Re–O chemical state is below the XPS detection limit after the UV-Ozone exposure. We estimate ~12% of S atoms on the surface are oxidized after UV-Ozone treatment, but the S/Re ratio remains identical to the initial substrate. Following the first pulse of TMA, the S–O chemical state falls below the XPS detection limit. Similar to the PEALD process, during the rest of the thermal ALD process the S–O chemical state stays under the XPS detection limit. After 80 cycles of TMA/H_2_O deposition, sulfur is below the XPS detection limit due to the signal attenuation of the deposited Al_2_O_3_. In addition, the Re 4*f* core level intensity is significantly decreased, making it difficult to deconvolve. Significant attenuation of Re 4*f* and S 2*p* core levels suggest that Al_2_O_3_ is comparable to the XPS depth of detection (~5 nm). The topographical AFM image of the Al_2_O_3_ film following 80 full cycles of Al_2_O_3_ deposition through the thermal ALD on a UV-Ozone pretreated ReS_2_ surface is shown in Figure 4b. The AFM image shows a conformal and pinhole free film with a very low RMS roughness of 0.11 nm, consistent with XPS analysis.

Figure 4c shows the evolution of the Al 2*p* and O 1*s* core level spectra. In the Al 2*p* core level, a peak is discernable at ~75 eV after the first TMA exposure, however, it is in the order of the XPS detection limit until the fifth TMA pulse. The O 1*s* core level again indicates the presence of two main components: an Al–O chemical state at low binding energy and an intermediate species chemical state at high binding energy. Utilizing the UV-Ozone pretreatment as a surface functionalization to deposit a thin Al_2_O_3_ film by thermal ALD produces the highest quality (conformal, pinhole-free) film on ReS_2_ in this work. The ex-situ Raman spectra of the ReS_2_ sample before and after being exposed to UV-Ozone at room temperature for 15 min and following 80 cycles of thermal ALD are shown in Figure 4d. Similar to the other processes, the Raman spectra did not show any changes after UV-Ozone exposure, demonstrating that the ReS_2_ structure stays intact.

Although both PEALD and the UV-Ozone pretreatment create S–O bonding on the surface, which serves as nucleation centers for subsequent ALD, the difference in the nucleation and Al_2_O_3_ growth behavior between the two methods is clear from AFM images. One important difference is that a higher S–O concentration was found for the UV-Ozone exposure in comparison to after the first O_2_ pulse in PEALD. This higher concentration of S–O bonds on the ReS_2_ surface helped with the pinhole issue of PEALD. Moreover, the concentration of the organic surface contamination after the UV-Ozone process is significantly less than after the PEALD process, as shown in the C 1*s* and O 1*s* XPS core level spectra (Figure S4). It is important to note that the prolonged X-ray exposure and UHV conditions occurred during the half-cycle XPS study do not change the surface chemistry of bulk ReS_2_ sample as indicated in Figure S5.

## 4. Conclusions

In summary, to achieve pinhole free and conformal high-κ dielectric on ReS_2_ substrate, we investigated different approaches for the growth of Al_2_O_3_. In-situ half-cycle XPS and ex-situ AFM were exploited to study the surface/interface chemistry and morphology of ReS_2_ after thermal ALD, PEALD, and UV-Ozone surface functionalization prior to ALD. Half-cycle XPS revealed that the ReS_2_ initial surface changes after the few first pulses of each process. The growth of Al_2_O_3_ by traditional ALD follows a substrate inhibited growth behavior, where an incubation period is required, followed by an islanding growth mode. However, the PEALD process enhances the growth of deposited Al_2_O_3_ by the creation of S–O bonds, but pinholes are still detectable even with lower RMS in comparison to the ALD growth. AFM images in conjunction with XPS data show that the UV-Ozone treatment is a practical route for the functionalization of ReS_2_ prior to ALD, to get a conformal coverage of Al_2_O_3_. Raman Spectroscopy and the XPS S/Re ratio confirms the structural stability of ReS_2_ under O_2_ plasma and UV-Ozone treatment.

## Supplementary Materials

The following are available online at https://www.mdpi.com/1996-1944/12/7/1056/s1, Figure S1: C 1*s*, O 1*s*, Re 4*f*, and S 2*p* core level spectra of initial ReS_2_ and subsequent annealing, Figure S2: In-situ half-cycle ALD/PEALD process, Figure S3: Wider XPS region of Al 2*p*, Section 4: XPS-calculated thickness, Figure S4: O 1*s* and C 1*s* core level spectra comparison, Figure S5: Re 4*f* and S 2*p* core level spectra of the exfoliated ReS_2_ sample after long x-ray exposure and being in UHV environment.
