# Supplementary material for: High-κ Dielectric on ReS2: In-Situ Thermal Versus Plasma-Enhanced Atomic Layer Deposition of Al2O3
Source: Materials (Basel). 2019 Mar 30;12(7):1056. doi: 10.3390/ma12071056 (PMC6479988; doi:10.3390/ma12071056)
Supplement: Supplementary file 1 [file materials-12-01056-s001.pdf]

materials

MDPI

Supplementary Materials

Supplementary Materials: High-κ Dielectric on ReS_2: In-situ Thermal Versus Plasma-Enhanced Atomic Layer Deposition of Al_2O_3

Ava Khosravi [1], Rafik Addou [1,2], Massimo Catalano [1,3], Jiyoung Kim [1] and Robert M. Wallace [1,*]

1. C 1s, O 1s, Re 4f, and S 2p Core Level Spectra of Initial ReS_2 and Subsequent Annealing

Figure S1 shows the C 1s, O 1s, Re 4f, and S 2p core levels obtained from the ReS_2 sample after exfoliation and subsequent annealing. It is noted from Figure S1a that the C 1s core level is convoluted with a loss feature of the Re $4d_{5/2}$ (261.7 eV) and Re $4d_{3/2}$ (275.5 eV). This loss feature has been seen in clean Re XPS spectra. Annealing the sample at 250 °C in UHV environment for one hour reduced the intensity of the C 1s signal below the XPS detection limit and also reduced adventitious oxygen chemical states; however, a small concentration (7 at.%) of oxygen remains on the surface (Figure S1b). The O 1s and C 1s core levels were detected from the initial surface of the ReS_2 sample likely originating from the environment after the ex-situ exfoliation process. Figure S1c,d shows Re 4f and S 2p core level spectra before and after annealing. No additional chemical states were detected in Re 4f and S 2p core level spectra after annealing revealing clearly that surface chemistry has not changed after annealing.

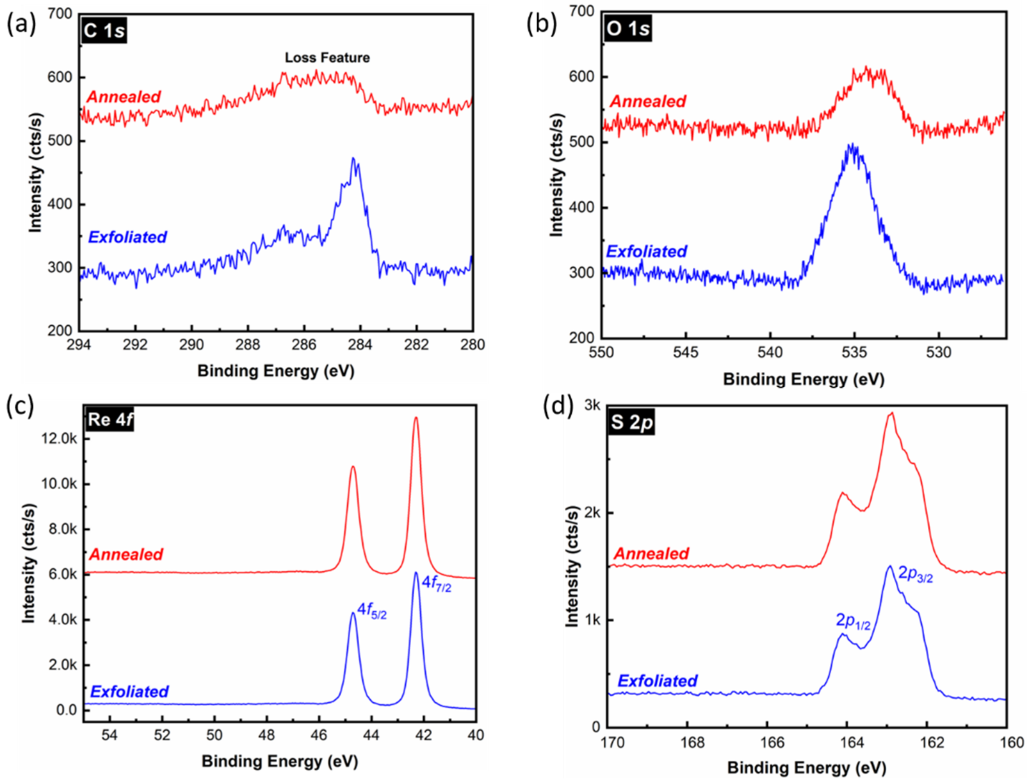

Figure S1. (**a**) C 1s, (**b**) O 1s, (**c**) Re 4f, and (**d**) S 2p core level spectra of initial ReS_2 and subsequent annealing for 1 h at 250 °C in UHV environment.

2. In-Situ Half-Cycle ALD/PEALD Process

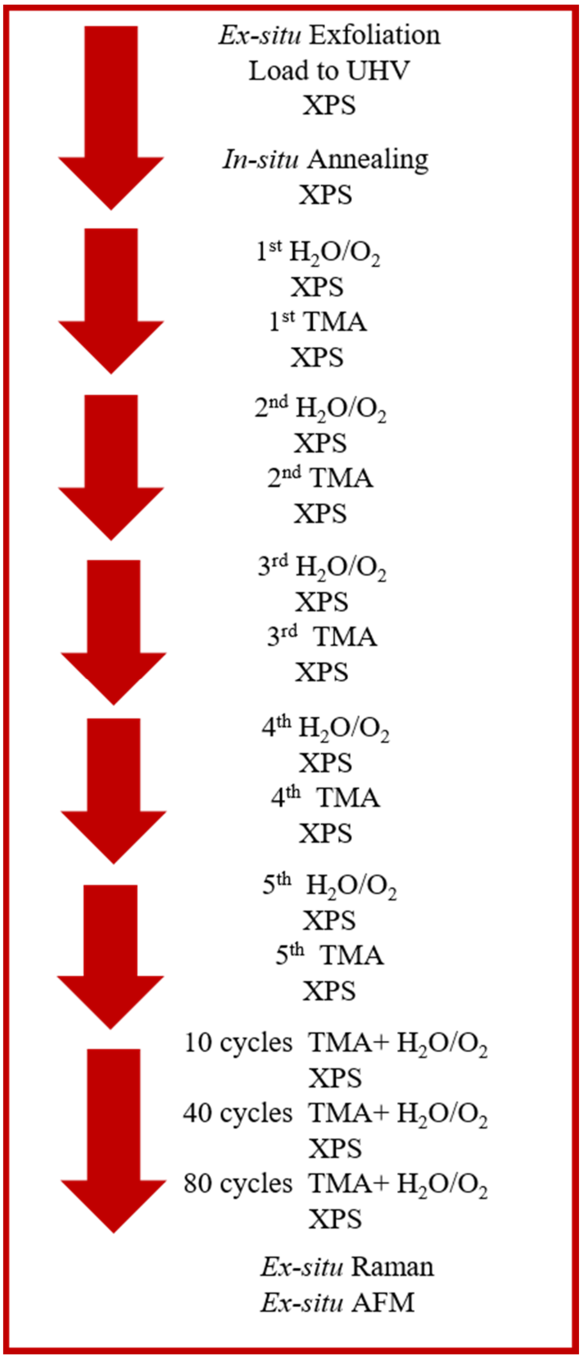

Figure S2. Half-cycle ALD/PEALD of Al_2O_3 on ReS_2.

3. Wider XPS Region of Al 2p

Figure S2 shows the Al 2p core level XPS spectrum recorded after 10 ALD cycles of Al_2O_3. This region is convoluted with a loss feature of Re 4f at 68 eV.

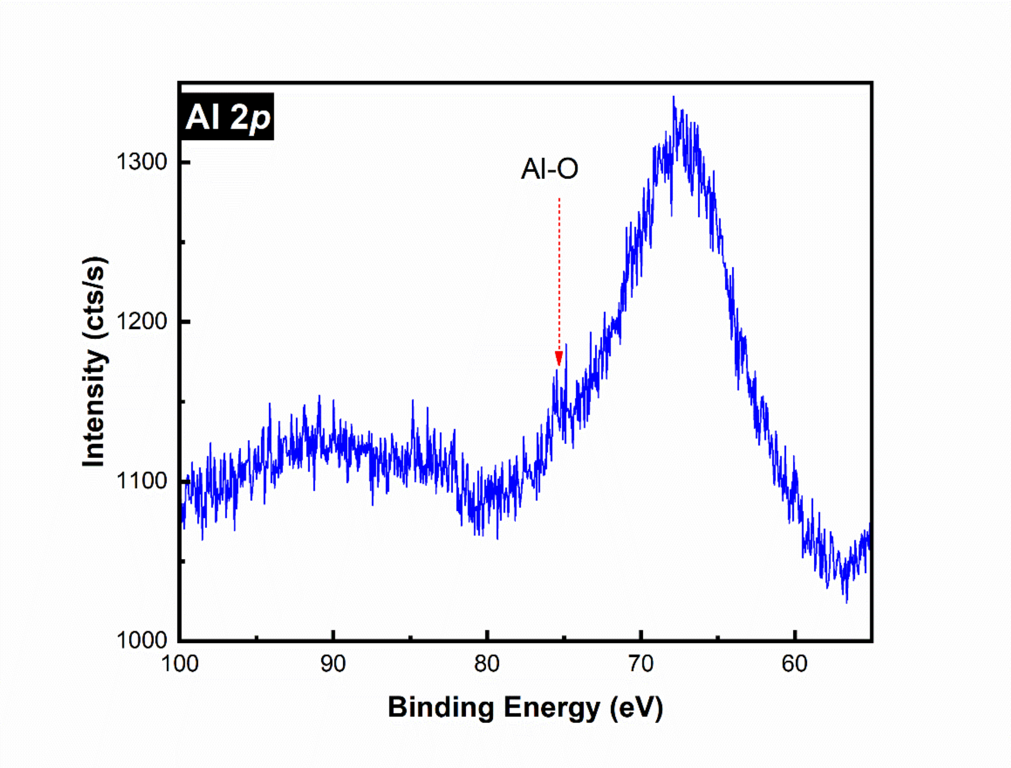

Figure S3. Al 2p core level spectrum acquired after 10 cycles ALD of ReS_2.

4. XPS-Calculated Thickness

The XPS-estimated Al_2O_3 thickness is calculated based on the attenuation of the substrate S 2p integrated intensity. The effective attenuation length (EAL) λ was calculated using the NIST electron EAL database, version 1.3, where $\lambda = 2.4$ nm for an electron travelling through amorphous Al_2O_3. The signl attenuation is calculated from:

$$I_s = I_s^0 e^{(-t/\lambda cos\theta)}$$

In this equation I_s is the attenuated electron signal, I_s^0 is the non-attenuated electron signal, t is the electron depth, λ is the inelastic mean free path, and θ is the XPS take-off angle (45°).

5. O 1*s* and C 1*s* Core Level Spectra Comparison

Figure S4 shows the C 1*s* and O 1*s* core level spectra after UV-Ozone treatment and after first O_2 pulse of PEALD process. After the first O_2 pulse in PEALD chamber, the C–O in both O 1*s* and C 1*s* core level spectra is significantly higher than that of UV-Ozone treated. Figure S4a demonstrates the additional chemical state at lower binding energy relates to the newly formed S-O bond. The intensity of S–O bonds created by UV-Ozone treatment is significantly higher than the first O_2 plasma pulse in PEALD process.

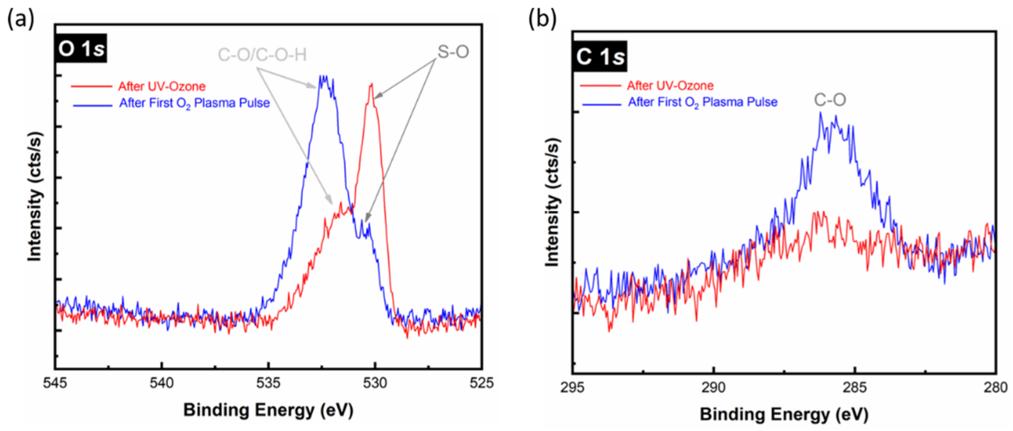

Figure S4. O 1s and C 1s core level spectra after UV-Ozone treatment and after first O_2 pulse in PEALD process.

6. Re 4f and S 2p Core Level Spectra after Long Time Under X-Ray Exposure and Being in UHV Environment

To monitor the changes on the ReS_2 surface chemistry under a prolonged time of X-ray exposure and UHV environment, as-exfoliated ReS_2 sample was exposed to X-rays for 3 h. All XPS scans were recorded in the exact same spot. Figure S5 shows the Re 4f and S 2p XPS core levels recorded on the as-exfoliated surface and following the X-rays exposure. XPS of the ReS_2 sample does not detect an additional chemical state or any changes on the surface chemistry after 3 h of X-rays exposure. The sample is then left in the UHV environment for 18 hours and a subsequent XPS was recorded on the same spot as shown in Figure S5a,b. Same as the prolonged X-ray exposure, no changes are detected on the surface chemistry of the sample. The prolonged X-ray exposure and UHV conditions occurred during the half-cycle study do not change the surface chemistry of ReS_2 sample. Our ex-situ Raman spectroscopy also confirms that the ReS_2 stays intact following all processing and acquisition conditions, as well as other half-cycle studies reported on different TMD surfaces. [1] Normalized spectra of Re 4f and S 2p core levels from as-exfoliated ReS_2 sample, after X-ray exposure, and after 18h in UHV are shown in Figure S5c,d.

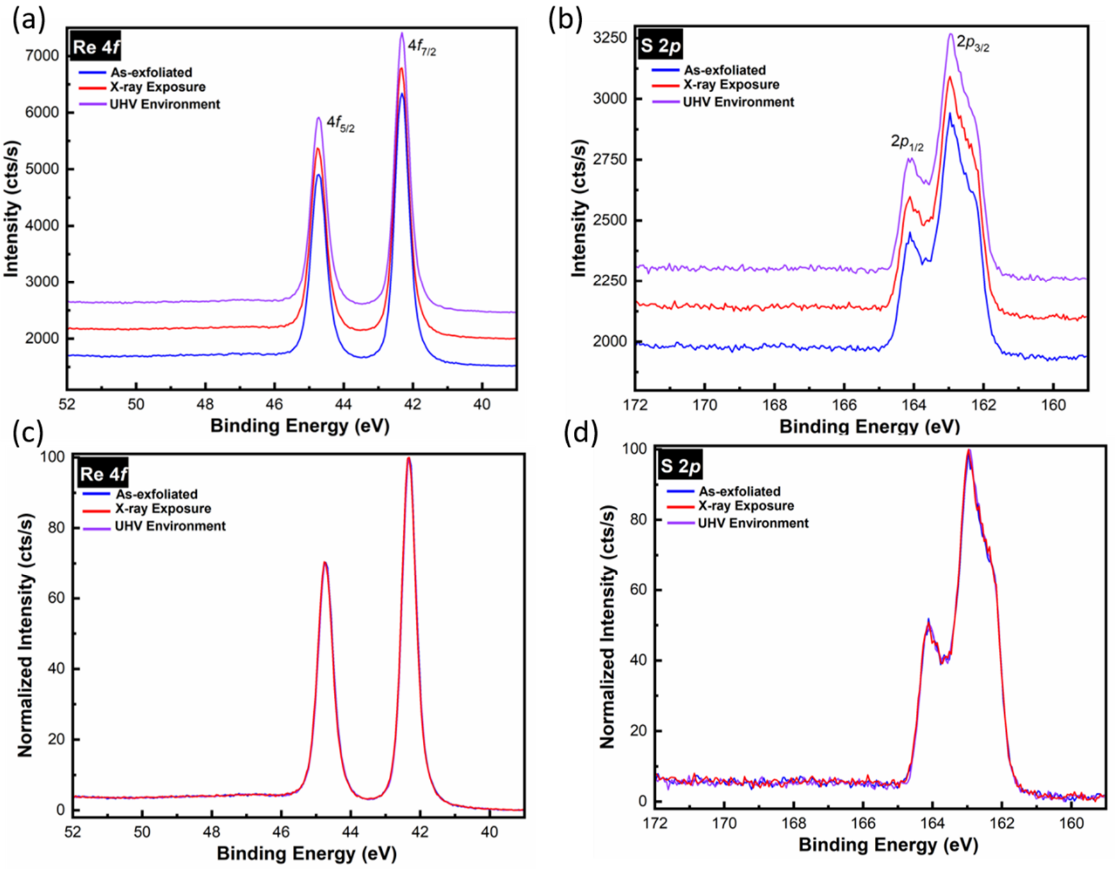

Figure S5. (**a**) Re 4f and (**b**) S 2p core level spectra from as-exfoliated ReS_2 (blue), after 3 hours X-ray exposure (Red), and after 18 hours in UHV environment (Purple). (**c**) Re 4f and (**d**) S 2p core level spectra with normalized intensity from as-exfoliated ReS_2 sample, after X-ray exposure, and after 18h in UHV.

References

1. McDonnell, S.; Brennan, B.; Azcatl, A.; Lu, N.; Dong, H.; Buie, C.; Kim. J.; Hinkle, C.L.; Kim, M. J.; Wallace, R.M. HfO_2 on MoS_2 by Atomic Layer Deposition: Adsorption Mechanisms and Thickness Scalability. *ACS Nano* **2013**, *7*, 10354.

© 2019 by the authors. Submitted for possible open access publication under the terms and conditions of the Creative Commons Attribution (CC BY) license (http://creativecommons.org/licenses/by/4.0/).
